# It Is Necessary to Re-understand the Low-Voltage Area in Atrial Fibrillation Patients

**DOI:** 10.3389/fcvm.2022.919873

**Published:** 2022-06-17

**Authors:** Wei Liu, Shijie Li, Bing Han

**Affiliations:** ^1^Bengbu Medical College, Bengbu, China; ^2^Department of Cardiology, Xuzhou Central Hospital, Xuzhou, China

**Keywords:** atrial fibrillation, atrial fibrosis, clinical characterization, low-voltage areas, atrial wall thickness

## Abstract

The presence of a low-voltage areas (LVAs) is a major feature of the progression of atrial fibrillation. Typically, the LVA is determined by invasive left atrial voltage mapping. In addition to pulmonary vein electrical isolation, Voltage-guided substrate modification by targeting LVAs in addition to PVI has been shown to be superior to conventional PVI “only” approaches regarding freedom from AF recurrences after ablation. Recent studies have found Atrial wall thickness correlates with low voltage areas, and the degree of atrial myocardial fibrosis can be better assessed by CT or MRI in combination with voltage mapping, which might help reduce the recurrence of AF after catheter ablation.

## What is the Low Voltage Area?

In patients with persistent AF, the incidence and size of LA low-voltage areas are higher than in patients with paroxysmal AF, and they are frequently found in the anterior left atrial wall, septum and posterior left atrial wall (1, 2). Most studies define the cutoff value for left atrial low voltage as a bipolar voltage ≤ 0.5 mV measured during sinus rhythm, and the low voltage zone is mainly located in the anterior, posterior, and interstitial walls. Recently, seveal studies have shown that it is feasible to conduct voltage measurements in atrial fibrillation rhythm, but the cutoff value differs from that of sinus rhythm, which was found to be <0.2 mV in the low voltage region when the left atrial voltage in conventional sinus rhythm was ≤ 0.5 mV as a reference standard (3). The presence of a low-voltage zone is associated with recurrent AF, stroke, and left atrial function after catheter ablation, and is more extensive in patients with persistent AF than in those with paroxysmal AF (2). A substantial amount of studies are known to suggest a correlation between atrial fibrosis and low voltage areas, and there is a high correlation between scarred areas identified by LGE-CMR and low voltage areas identified by electroanatomical calibrations. Electro-mapping might predict atrial fibrosis (4).

## Low Voltage Areas do Not Absolutely Reflect Atrial Myocardial Fibrosis

The low voltage region does not absolutely reflect the degree of atrial fibrosis. A few influencing factors including different atrial rhythms, atrial wall thickness and contact area between the recording catheter and the atrial wall, conduction velocity, fiber alignment, and recording catheter characteristics (electrode size, spacing, and orientation with respect to the tissue) can affect voltage labeling, which has been demonstrated in animal and human studies. Andrés et al. reported a wider range of low-voltage areas scaled under atrial fibrillation rhythm (AF) than in sinus rhythm (SR) (5).

## Effect of Atrial Thickness on Electroanatomical Mapping

In addition to reflecting atrial muscle fibrosis in the low-voltage area of mapping, we also need to consider the influence of atrial muscle thickness on the mapping results. Recent studies have shown evidence that changes in left atrial myocardial thickness are an important part of this pathological remodeling process. Autopsy and imaging studies have demonstrated changes in atrial wall thickness between patients with different clinical histories. Combined with atrial tissue characterization, a comprehensive assessment of atrial structure might help predict atrial electrophysiological behavior and guide regional RF treatment based on atrial wall thickness. Platonov et al. observed (6) the posterior wall of the left atrium in 298 consecutive pathological specimens in routine autopsy. In this study, patients with a history of atrial fibrillation had significantly thinner posterior left atrial walls compared with patients without a history of atrial fibrillation. In the present study, the mean posterior wall thickness in the middle and lower parts of the posterior wall in the non-AF group was 2.6 and 2.9 mm, respectively, and the mean posterior wall thickness at each segment in the AF group was 0.4 mm. Atrial wall thickness is likely to be a useful marker of atrial pathological remodeling in patients at risk for AF or in patients already diagnosed with AF.

Studies have shown that atrial muscle thickness is closely related to low-voltage areas. For example, Wi et al. (7) confirmed the relationship between atrial wall thickness and fragmentation potential measured by computed tomography (CT). Takahashi et al. (8) also demonstrated that the thinning of the pulmonary vein-atrial junction was accompanied by a decrease in bipolar voltage, and the pulmonary vein-atrial junction correlated with bipolar voltage amplitude. Nakatani et al. (9) suggested that left atrial wall thickness is a predictor of reconnection and resting conduction after pulmonary vein electrical isolation. Recently, Nakatani et al. confirmed (10) that atrial wall thickness correlates with low voltage areas. Atrial wall thickness might reflects atrial remodeling, and atrial wall thickness is also related to the local reduced potential of atrial tissue. Atrial wall thickness reflects atrial remodeling, and atrial wall thickness is also associated with locally reduced potentials in atrial tissue, and the authors suggest that atrial wall thickness may change as atrial fibrillation progresses. Atrial wall thickness is thicker in patients with paroxysmal atrial fibrillation than in healthy individuals. The atrial wall thickness is thinner in persistent AF than in paroxysmal AF, probably because the increased atrial pressure and atrial volume reduces the atrial wall thickness. In this study, atrial wall thickness assessed by contrast-enhanced CT correlated with low-voltage areas of atrial tissue measured by three-dimensional mapping in 43 patients with paroxysmal atrial fibrillation who underwent first catheter ablation and underwent electrical mapping. Preoperative multi-slice spiral CT was used to measure the thickness of the atrial wall (excluding fat) of the research subjects, and the left atrium was artificially divided into 21 parts, including the septal wall (3 parts), the anterior wall of the left atrium (3 parts), and the apex. Wall (3 parts), bottom wall (3 parts), rear wall (9 parts). It was confirmed that the thickness of different parts of the left atrium varies greatly, and the thickness of the septal wall is the thinnest compared with other parts. In addition, the anterior and the roof walls of the atrium are significantly thicker than the posterior and bottom walls. The division of the left atrial 3D reconstruction area by electrical mapping is consistent with the method of CT division. In this study, a correlation analysis was performed between the atrial thickness and the low-voltage area measured by the electrical mapping performed during the operation, and the univariate linear regression obtained the left atrium. Wall thickness, left atrial volume, and left atrial appendage blood flow velocity were significantly associated with left atrial low-voltage areas. Multivariate linear regression showed that left atrial wall thickness and left atrial volume were independent determinants of the low voltage area of the left atrium (*P* = 0.005, *P* = 0.002). Myocardial thickness at 215 sites in 43 patients was divided into 3 groups: low, medium and high. Compared with the medium thickness group (1.76–2.14 mm) and the high thickness group (≥2.14 mm), the myocardial thickness in the low thickness group (<1.76 mm) was lower. The presence of voltage regions is more extensive. Therefore, the thin left atrium can map a larger area of low voltage. Through ROC curve analysis, the atrial wall below 1.9 mm can predict a wider low-voltage area, that is, the thinner the left atrial wall thickness, the wider the degree and scope of the atrial low-voltage area. Based on the study, the authors concluded that regions of the atrial wall smaller than 1.9 mm require accurate voltage mapping. Therefore, we can predict the extent and extent of the low-voltage zone by measuring the thickness of the atrial wall.

There are also some studies showing that atrial muscle thickness can predict the recurrence of atrial fibrillation, and three-dimensional mapping of low-voltage areas has certain limitations compared with imaging. Nakatani et al. (10) also evaluated the effect of atrial muscle thickness on the recurrence of atrial fibrillation by evaluating the thickness of the atrial wall in each region by contrast-enhanced CT. The study included 128 patients with paroxysmal atrial fibrillation and 85 patients with persistent atrial fibrillation. The atrial wall thickness in each region was assessed by contrast-enhanced CT. Compared with the non-Af recurrence group, the Af recurrence group had thicker atrial walls and the coefficient of variation of wall thickness was larger, which inferred that atrial wall thickness could predict AF recurrence. However, voltage mapping has its own limitations, representing only tissue features within a few millimeters of the catheter tip, limiting their sensitivity to detect fibrosis and scarring in the middle of the myocardium. Kucukseymen et al. (11) obtained inconsistencies between delayed enhancement scar regions and low-voltage regions detected by 3D mapping through animal experiments, highlighting the limitations of current 3D mapping systems in detecting scars in thick-walled myocardial wall regions. The authors concluded that in AF patients with thicker atrial walls, the mapping of low-voltage areas was affected by atrial wall thickness. Because the area with thicker atrial wall has fewer low-voltage areas detected by mapping than the area with thinner atrial wall, less matrix modification is performed on the thicker atrial wall, so the recurrence rate of atrial fibrillation is affected by this aspect.

So does the thinner the myocardial thickness, the larger the left atrial volume? Nakatani et al. also compared the atrial wall thickness of patients with paroxysmal Af and persistent Af, and concluded that the atrial wall thickness in patients with paroxysmal Af is thicker than that in persistent Af. Notably, this study also concluded that there was no correlation between LA wall thickness and LA volume, and concluded that the hypothesis of LA wall thinning due to LA volume pressure overload is not valid. The analysis suggested that the thinning of the left atrial wall in persistent atrial fibrillation may be the result of structural remodeling. During Af, rapid atrial activation caused relative ischemia and oxidative stress in the atrial myocardium, which could lead to apoptosis and necrosis of atrial myocytes. Decreased myocardial mass and reactive interstitial fibrosis may reduce local atrial wall thickness and potential amplitude. Seewöster et al. (12) report that the relationship between left atrial volume and left atrial fibrosis was rather unpredictable, as some patients had extensive fibrosis but normal left atrial volume. Therefore, it is necessary to investigate whether there is a correlation between atrial wall thickness and left atrial volume.

## Summary and Clinical Outlook

At present, it is generally believed that low-voltage area is more extensive in patients with the persistent Af, and the ablation effect of the low-voltage area in patients with persistent atrial fibrillation is more significant than that of pulmonary vein electrical isolation alone (13). Different studies on the degree of left atrial fibrosis have drawn different conclusions. Some people with paroxysmal AF have severe fibrosis, while others with persistent AF have less fibrosis. Therefore, preoperative CT or MR examination can be used to more systematically and comprehensively identify the atrial muscle thickness, degree of atrial muscle fibrosis, and the relationship between the thickness of the left atrial wall and the low-voltage area in patients with atrial fibrillation, reducing the need for intraoperative three-dimensional markers. It is of great significance to prevent the misjudgment of measurement, reduce postoperative recurrence, and formulate more accurate ablation strategies for atrial fibrillation.

## Author Contributions

WL: writing-original draft. SL: conceptualization, funding acquisition, resources, supervision, and writing-review and editing. BH: supervision. All authors contributed to the article and approved the submitted version.

## Conflict of Interest

The authors declare that the research was conducted in the absence of any commercial or financial relationships that could be construed as a potential conflict of interest.

## Publisher's Note

All claims expressed in this article are solely those of the authors and do not necessarily represent those of their affiliated organizations, or those of the publisher, the editors and the reviewers. Any product that may be evaluated in this article, or claim that may be made by its manufacturer, is not guaranteed or endorsed by the publisher.
